# Overview of Meta-Analyses of Five Non-pharmacological Interventions for Alzheimer's Disease

**DOI:** 10.3389/fnagi.2020.594432

**Published:** 2020-11-25

**Authors:** Liao-Yao Wang, Jian Pei, Yi-Jun Zhan, Yi-Wen Cai

**Affiliations:** Department of Acupuncture, Longhua Hospital, Shanghai University of Traditional Chinese Medicine, Shanghai, China

**Keywords:** Alzheheimer's disease, non-pharmacological intervention, overview, meta–analysis, effectiveness

## Abstract

**Background:** Alzheimer's disease (AD) is a neurodegenerative disease characterized by progressive memory deficits, cognitive decline, and spatial disorientation. Non-pharmacological interventions to treat AD have been reported in many meta-analyses (MAs), but robust conclusions have not been made because of variations in the scope, quality, and findings of these reviews.

**Objective:** This work aimed to review existing MAs to provide an overview of existing evidence on the effects of five non-pharmacological interventions in AD patients on three outcomes: Mini-Mental State Examination (MMSE), activities of daily living (ADL), and Alzheimer's Disease Assessment Scale-cognitive section (ADAS-cog).

**Methods:** The databases PubMed, Cochrane Library, Embase, and Web of Science were searched to collect MAs of non-pharmacological interventions for AD. Two reviewers independently conducted literature screening, data extraction, and quality assessment. We assessed the quality of MAs with the Measurement Tool to Assess Systematic Reviews (AMSTAR) 2 and assessed the evidence quality for significant outcomes using the Grading of Recommendations Assessment, Development, and Evaluation (GRADE) system.

**Results:** We found 10 eligible MAs, which included between three (133 patients) and 15 randomized trials (1,217 patients), and five non-pharmacological interventions, namely, acupuncture therapy (40%), exercise intervention (30%), music therapy (10%), cognitive intervention (10%), and repetitive transcranial magnetic stimulation (rTMS) (10%). All the included MAs were critically low to low quality by AMSTAR 2. Acupuncture therapy and exercise intervention showed the preliminary potential to improve ADL and MMSE. rTMS and acupuncture therapy show benefits in decreasing ADAS-cog, and there were some evidence of improved MMSE with cognitive intervention. All these outcomes scored very low quality to moderate quality of evidence on the GRADE system.

**Conclusions:** Non-pharmacological therapy shows promise for the treatment of AD, but there is still a lack of high-quality evidence. In the future, the quality of the original research needs to be improved, and strictly designed MAs should be carried out following methodological requirements.

## Introduction

Dementia is a common public health problem globally, affecting approximately 47 million people worldwide, and is expected to increase to 131.5 million by 2050 (Alzheimer's Disease International, 2015; Arvanitakis et al., 2019). Alzheimer's disease (AD) is a neurodegenerative disease characterized by progressive memory deficits, cognitive decline, and spatial disorientation. Among all types of dementia, AD accounts for 60–80%. An estimated 5.8 million Americans aged 65 and older are living with AD in 2020 (Alzheimer's Association, 2020). The incidence of AD in China was reported as 6.25 cases per 1,000 person-years, and the median standardized mortality ratio was 1.94:1 (Chan et al., 2013). With the progressive death of neurons and damage to brain tissue, AD becomes more severe over time. The progression of AD will lead to problems with memory and, eventually, physical disability (Alzheimer's Association, 2020). AD patients also often have other concomitant diseases that may exacerbate progression and symptoms (Haapasalo et al., 2015). AD is officially listed as the sixth leading cause of death (Xu et al., 2020). With the aging of populations, the high cost of AD imposes an increasingly heavy burden worldwide on patients, family members, healthcare systems, and society (Maresova et al., 2018; Pedroza et al., 2019).

Currently, the main pharmacotherapeutic options for AD are cholinesterase inhibitors (donepezil, rivastigmine, and galantamine) and memantine (O'Brien et al., 2017). However, none of these can slow down or stop the damage and destruction of neurons or the progression of the disease (Alzheimer's Association, 2020). Therefore, more potential and effective treatments need to be assessed. In recent years, increasing attention has been focused on complementary and alternative therapies for AD; among them, non-pharmacological therapies play an important complementary role (Olazaran et al., 2010; Zucchella et al., 2018).

Meta-analyses (MAs) have been conducted to summarize the efficacy of different types of non-pharmacologic therapies for AD (García-Casares et al., 2017; Cammisuli et al., 2018; Sá et al., 2019). However, strong conclusions have not been reached owing to the wide variation in scope, quality, and outcomes. Although study (Kishita et al., 2020) has shown that non-pharmacological interventions improve depression, anxiety, and quality of life in people with dementia, they did not identify the different types of dementia. Therefore, as an overview, we evaluated the quality of MAs of non-pharmacological therapies and summarized the evidence on their effects on the Mini-Mental State Examination (MMSE), activities of daily living (ADL), and Alzheimer's Disease Assessment Scale-cognitive section (ADAS-cog) outcomes in patients with AD.

## Methods

### Protocol and Registration

We undertook an overview of MAs in line with Preferred Reporting Items for Overview of systematic reviews (Bougioukas et al., 2018), together with the prospective protocol registered in INPLASY (registration no. INPLASY202070014). Ethics approval was not required.

### Literature Search

A comprehensive search of four databases comprising PubMed, Cochrane Library, Embase, and Web of Science was undertaken up to 15 May 2020 for MAs evaluating non-pharmacological interventions currently used in AD patients. References from related articles were also searched. There were no language restrictions. The details of the search strategy are given in Supplementary Material 1.

### Inclusion Criteria

The inclusion criteria are: (1) population: participants diagnosed as AD; (2) intervention: specific non-pharmacological intervention including, but not limited to, acupuncture therapy, music therapy, exercise interventions, *etc*., was performed alone or in combination with other treatments; (3) control: medication or placebo or another type of treatment corresponding to the intervention was applied as a comparison; (4) outcome: outcome measures were quantitatively reported as MMSE, ADL, or ADAS-cog; and (5) study: MA of randomized clinical trials (RCTs).

The participants were diagnosed as AD by common criteria such as the National Institute of Neurological and Communicative Disorders and Stroke Alzheimer's Disease and Related Disorders Association (McKhann et al., 1984; Dubois et al., 2007), or it was clearly stated that the subjects of the study were AD patients.

### Exclusion Criteria

The exclusion criteria included: (1) AD patients combined with other diseases and mixed samples (AD participants and other types of dementia, mild cognitive impairment, or other related neurocognitive disorders), (2) immunotherapies (vaccine, monoclonal antibodies) and nutritional components (nutraceuticals) administered, (3) systematic reviews (SRs) without MAs of outcomes, (4) SRs with network MAs or Bayesian MAs, and (5) protocols, meeting abstracts, and MAs without full text.

### Study Selection

All retrieved studies were imported into Endnote X9 software, and duplicate results were deleted. Based on the selection criteria, two independent researchers (Liaoyao Wang, Yijun Zhan) selected the relevant studies after screening titles and abstracts. Inter-assessor discrepancies would be resolved by discussion or arbitration of a third reviewer (Jian Pei).

The full text of all potential articles was obtained for a detailed evaluation according to the inclusion/exclusion criteria, and the final relevant studies were shortlisted. Any disagreement was resolved by a discussion with a third reviewer (Jian Pei) or by final group consensus.

### Data Extraction

A standard form was used for data extraction from all MAs, containing the following: year of publication, first author, database, number of included trials and participants, type of interventions, outcomes, quality assessment tools, main findings, *etc*. Data were extracted by two independent reviewers (Yiwen Cai, Yijun Zhan). Any discrepancies were resolved by consensus after all the authors re-reviewed the study.

### Assessment of Methodological Quality

We used A Measurement Tool to Assess Systematic Reviews 2 (AMSTAR 2) (Shea et al., 2017) to assess the methodological quality of the included MAs. AMSTAR 2 is a comprehensive critical appraisal tool for SRs of randomized and non-randomized studies that have simpler response categories than the original AMSTAR and, more importantly, is not intended to generate an overall score but instead focuses on weaknesses in critical domains (Shea et al., 2017). A psychometric study found that AMSTAR 2 is a valid and moderately reliable appraisal tool (Lorenz et al., 2019). The process of assessment was independently performed by two of the authors, and any discrepancies were resolved by final consensus among all the authors. AMSTAR 2 has 16 items in total, each of which is evaluated as “yes,” “partial yes,” or “no.” Seven items (2, 4, 7, 9, 11, 13, and 15) were considered as critical domains that critically affected the validity of a review and its conclusions. Overall confidence in the results of the review was rated as “high” (none or one non-critical weakness), “moderate” (more than one non-critical weakness but no critical flaws), “low” (one critical flaw ± non-critical weaknesses), and “critically low” (more than one critical flaw ± non-critical weaknesses) (Shea et al., 2017). The details of the 16 domains of AMSTAR 2 are shown in Supplementary Material 2.

### Assessment of Evidence Quality

The Grading of Recommendation, Assessment, Development, and Evaluation (GRADE) (Guyatt et al., 2008) system was used to assess the evidence quality for each outcome on four degrees (high, moderate, low, and very low quality) by two authors independently. There are now more than 110 organizations from 19 countries that have endorsed or use GRADE to judge the quality of evidence bearing on clinical questions and to develop corresponding clinical practice guidelines (Gordon and Guyatt, 2020). We used the GRADE assessment of evidence quality, with the following criteria. For each significant outcome, we initially awarded high quality because these were based on RCTs. We then downgraded the confidence according to five aspects: risk of bias, inconsistency, indirectness, imprecision, and publication bias. A final consensus among all the authors resolved any discrepancies among two of the authors.

## Results

### Study Selection Process

**Figure 1:**
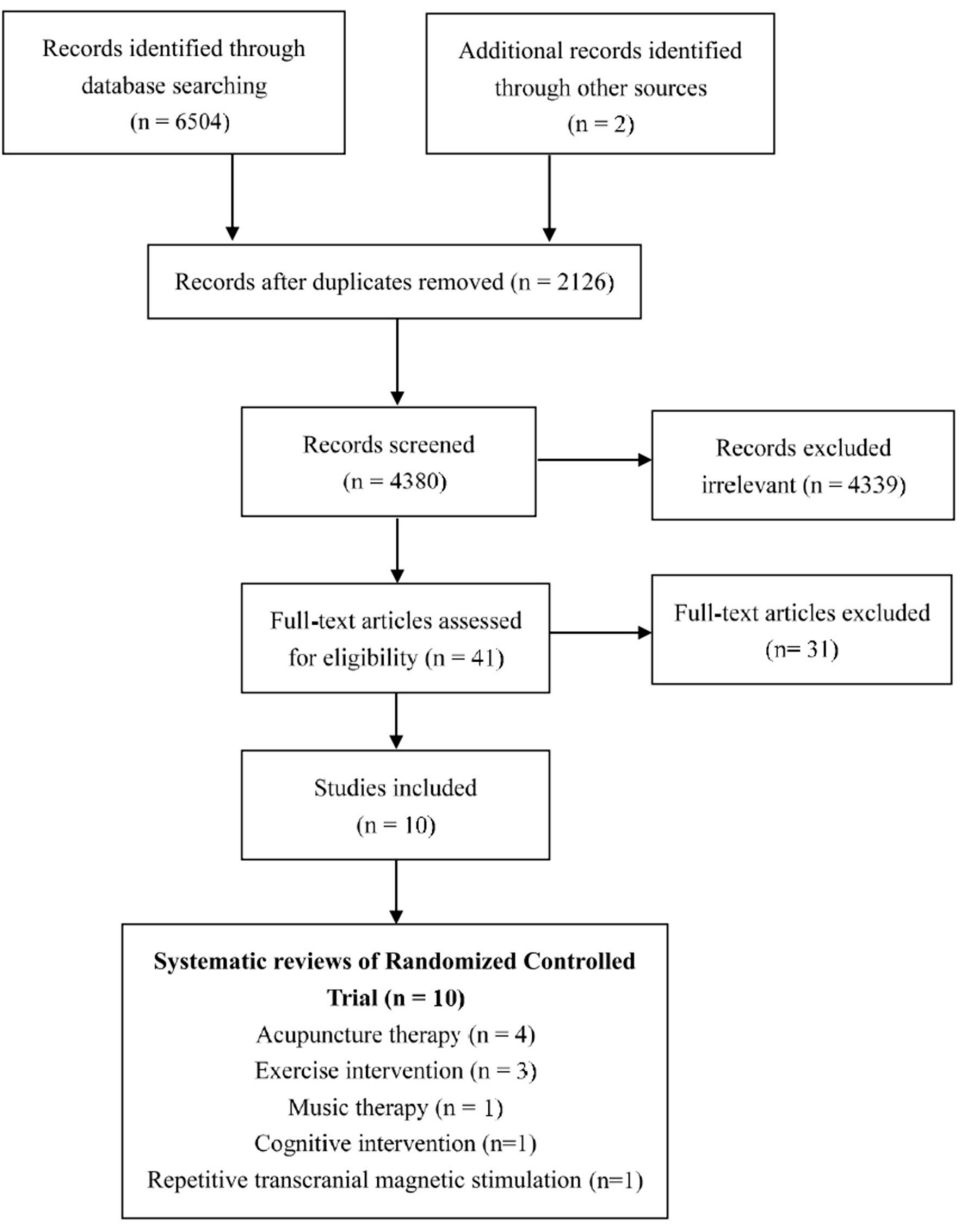
Study selection process.

The searches identified 6,504 published studies, and after screening the titles and removing duplicates, 41 potentially eligible studies were selected for a closer scrutiny by retrieving the full text. Finally, we included 10 MAs (Lee et al., 2009; Alves et al., 2013; Rao et al., 2014; Zhou et al., 2015, 2017; Dong et al., 2018; Du et al., 2018; Huang et al., 2019; Jia et al., 2019; Wang et al., 2020). The study selection process is shown in Figure 1.

### Characteristics of the Included Studies

The 10 selected MAs reported 3–15 RCTs with 133–1,217 AD patients. Four MAs (Lee et al., 2009; Zhou et al., 2015, 2017; Huang et al., 2019) assessed the effects of acupuncture therapy, three (Rao et al., 2014; Du et al., 2018; Jia et al., 2019) assessed exercise (aerobic exercise/physical activity/cycle ergometer exercise/brisk walking, *etc*.), one (Alves et al., 2013) assessed cognitive stimulation therapy (memory-training program/attention-stimulating activities/cognitive training), one (Wang et al., 2020) assessed music therapy, and one (Dong et al., 2018) evaluated repetitive transcranial magnetic stimulation (rTMS). The details of the included MAs are shown in Table 1, and the specific type, content, intensity, and duration of each of the five interventions evaluated in these MAs are shown in Supplementary Material 3.

**Table 1:** Characteristics of the included meta-analyses.

**References**	**Number of databases searched**	***N*/*n***	**Severity of Alzheimers disease (AD)**	**Intervention**	**Outcomes**	**Side effect**	**Main findings**	**AMSTAR 2**
Lee et al. (2009)	20	3 (166)	Mild to moderate (*N* = 2); N.R. (*N* = 1)	Acupuncture therapy	MMSE, ADL	N.R.	“Existing evidence does not demonstrate the effectiveness of acupuncture for AD.” (Lee et al., 2009)	Critically low
Zhou et al. (2017)	11	15 (1,217)	N.R.	Acupuncture therapy	MMSE, ADL, ADAS-cog	No	“Acupuncture plus Chinese medicine may have advantages over Western drugs for treating AD.” (Zhou et al., 2017)	Low
Zhou et al. (2015)	8	10 (585)	N.R.	Acupuncture therapy	MMSE, ADL, ADAS-cog	No	“Acupuncture may be more effective than drugs and may enhance the effect of drugs for treating AD in terms of improving cognitive function. Acupuncture may also be more effective than drugs at improving AD patients' ability to carry out ADLs. Moreover, acupuncture is safe for treating people with AD.” (Zhou et al., 2015)	Critically low
Huang et al. (2019)	8	13 (750)	N.R.	Acupuncture therapy	MMSE, ADL, ADAS-cog	Yes	“Acupuncture alone was better than conventional Western medicines for the treatment of AD.” (Huang et al., 2019)	Low
Jia et al. (2019)	5	13 (673)	Moderate (*N* = 12); Moderate to high (*N* = 1)	Exercise intervention	MMSE	Yes	“Physical activity and exercise can improve cognition of older adults with AD. While the concomitant effects on cognition of high frequency interventions was not greater than that of low frequency interventions, the threshold remains to be settled.” (Jia et al., 2019)	Critically low
Alves et al. (2013)	11	4 (133)	N.R.	Cognitive stimulation therapy	MMSE	N.R.	“Results demonstrate absence of effects of cognitive intervention in most of the analyzed domains and evidence of cognitive intervention effects toward improvement in global cognitive functioning as measured by MMSE.” (Alves et al., 2013)	Critically low
Wang et al. (2020)	5	15 (765)	N.R.	Music therapy	MMSE, ADL	N.R.	“The effect of music therapy on cognitive function and ADL in patients with AD is not significant.” (Wang et al., 2020)	Critically low
Dong et al. (2018)	3	5 (148)	N.R.	Repetitive transcranial magnetic stimulation (rTMS)	MMSE, ADAS-cog	Yes	“rTMS is relatively well-tolerated, with some promise for cognitive improvement and global impression in patients with AD. Our findings also indicate the variability between ADAS-cog and MMSE in evaluating global cognitive impairment.” (Dong et al., 2018)	Low
Rao et al. (2014)	6	6 (446)	N.R.	Exercise intervention	ADL	Yes	“Occupational therapy intervention that includes aerobic and strengthening exercises may help improve independence in ADLs and improve physical performance in people with AD.” (Rao et al., 2014)	Critically low
Du et al. (2018)	8	13 (869)	N.R.	Exercise intervention	MMSE	N.R.	“This meta-analysis and systematic review indicated that exercise intervention might improve the cognitive function of AD or slow down the decline of cognition; however, this relationship was not always true across studies.” (Du et al., 2018)	Critically low

*N: No. of RCTs; n: No. of Participants; N.R.: Not Reported*.

### Methodological Quality of the Included MAs

**Figure 2:**
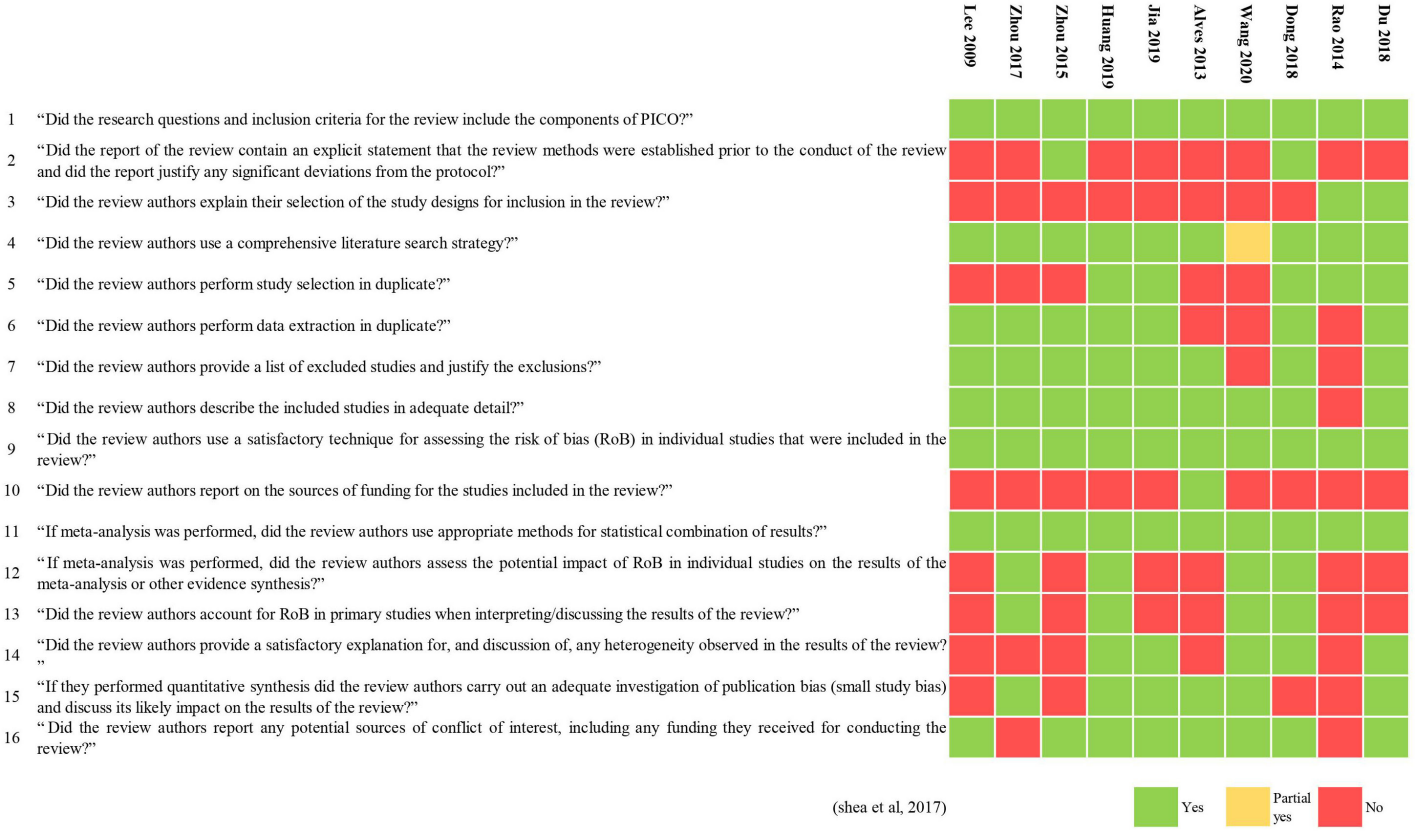
Evaluation results of the included meta-analyses by AMSTAR 2.

The methodological quality of three MAs (Zhou et al., 2017; Dong et al., 2018; Huang et al., 2019) was low, and that of all the others was critically low. All included MAs specified their inclusion and exclusion criteria, including PICO (population, interventions, comparators, and outcomes), undertook a comprehensive literature search, used a useful technique for assessing the risk of bias, and used appropriate methods for the statistical combination of results. Only two reviews (Zhou et al., 2015; Dong et al., 2018) had registered and had a protocol before performing the review. Six MAs (Alves et al., 2013; Zhou et al., 2017; Du et al., 2018; Huang et al., 2019; Jia et al., 2019; Wang et al., 2020) discussed publication bias. The results of the evaluation by AMSTAR 2 are shown in Table 1 and Figure 2.

### Evidence Quality of Outcomes

This overview focused on three outcomes: MMSE, ADL, and ADAS-cog. The evidence synthesis for each outcome by the GRADE system is summarized below and in Table 2. There were 10 (41.67%) moderate-quality evidences, 11 (45.83%) low-quality evidences, and three (12.50%) very low-quality evidence.

**Table 2:** Quality of evidence by Grading of Recommendations Assessment, Development, and Evaluation (GRADE) system.

**References**	**Intervention**	**Outcomes**	**Number of study**	**Confidence interval (95%)**	**GRADE**					**Evidence quality**
					**Risk of bias**	**Inconsistency**	**Indirectness**	**Imprecision**	**Publication bias**	
Lee et al. (2009)	Acupuncture vs. drugs	MMSE	2	MD −0.55 (−1.31, 0.21)	−1	0	0	−1	0	Low
		ADL	2	MD −1.29 (−1.77, −0.80)	−1	−1	0	−1	0	Very low
Zhou et al. (2015)	Acupuncture vs. drugs	MMSE	6	MD 1.05 (0.16, 1.93)	−1	−1	0	−1	0	Very low
		ADL	4	MD −2.80 (−4.57, −1.02)	−1	0	0	−1	0	Low
		ADAS-cog	1	MD −5.14 (−8.75, −1.53)	0	/	0	−1	0	Moderate
	Acupuncture + drugs vs. drugs	MMSE	3	MD 2.37 (1.53, 3.21)	−1	0	0	−1	0	Low
		ADL	2	MD −2.64 (−4.95, −0.32)	−1	0	0	−1	0	Low
		ADAS-cog	1	MD −0.90 (−4.00, 2.20)	0	/	0	−1	0	Moderate
	Acupuncture vs. no treatment	MMSE	1	MD 3.74 (1.34, 6.14)	−1	/	0	−1	0	Low
		ADL	1	MD −8.82 (−19.83, 2.19)	−1	/	0	−1	0	Low
Zhou et al. (2017)	Acupuncture + drugs vs. drugs	MMSE	11	MD 1.41 (0.97, 1.85)	0	0	0	0	−1	Moderate
		ADL	3	MD −3.59 (−7.18, 0.01)	0	−1	0	−1	0	Low
		ADAS-cog	1	MD −0.03 (−1.71, 1.65)	0	/	0	−1	0	Moderate
Jia et al. (2019)	Exercise intervention	MMSE	13	SMD 1.12 (0.66, 1.59)	0	−1	0	0	0	Moderate
Alves et al. (2013)	Cognitive intervention	MMSE	3	MD 0.87 (0.26, 1.48)	0	−1	0	−1	0	Low
Huang et al. (2019)	Acupuncture vs. drugs	MMSE	10	WMD 1.96 (0.66, 3.26)	0	−1	0	0	0	Moderate
		ADL	8	WMD 1.99 (0.65, 3.34)	0	−1	0	0	0	Moderate
		ADAS-cog	3	WMD 5.56 (1.10, 6.03)	0	−1	0	−1	0	Low
Wang et al. (2020)	Music therapy	MMSE	15	SMD 0.14 (−0.36, 0.63)	−1	−1	0	0	0	Low
		ADL	4	SMD −0.03 (−0.23, 0.17)	−1	−1	0	0	0	Low
Dong et al. (2018)	rTMS	MMSE	4	MD 0.32 (−1.30, 1.94)	0	0	0	−1	0	Moderate
		ADAS-cog	3	MD −3.65 (−5.82, −1.48)	0	0	0	−1	0	Moderate
Rao et al. (2014)	Exercise intervention	ADL	6	MD 0.80 (0.42, 1.19)	−1	−1	0	−1	−1	Very low
Du et al. (2018)	Exercise intervention	MMSE	7	MD 2.53 (0.84, 4.22)	0	−1	0	0	0	Moderate

#### MMSE

Nine MAs (Lee et al., 2009; Alves et al., 2013; Zhou et al., 2015, 2017; Dong et al., 2018; Du et al., 2018; Huang et al., 2019; Jia et al., 2019; Wang et al., 2020) reported the MMSE. Three of the interventions (acupuncture therapy, exercise, and cognitive intervention) improved MMSE significantly. Acupuncture vs. drugs (MD = 1.05, 95% CI: 0.16, 1.93; very low quality; Zhou et al., 2015; WMD = 1.96, 95% CI: 0.66, 3.26; moderate-quality; Huang et al., 2019) and acupuncture vs. no treatment (MD = 3.74, 95% CI: 1.34, 6.14; low quality; Zhou et al., 2015) improved the MMSE. A combination of acupuncture and drugs vs. drugs alone (MD = 2.37, 95% CI: 1.53, 3.21; low quality; Zhou et al., 2015; MD = 1.41, 95% CI: 0.97, 1.85; moderate-quality; Zhou et al., 2017) improved the MMSE. Exercise intervention (SMD = 1.12, 95% CI: 0.66, 1.59; moderate-quality; Jia et al., 2019; MD = 2.53, 95% CI: 0.84, 4.22; moderate-quality; Du et al., 2018) and cognitive intervention (MD = 0.87, 95% CI: 0.26, 1.48; low quality) (Alves et al., 2013) significantly improved the MMSE.

#### ADL

Six MAs (Lee et al., 2009; Rao et al., 2014; Zhou et al., 2015, 2017; Huang et al., 2019; Wang et al., 2020) reported on ADL. Acupuncture and exercise intervention demonstrated a significant effect on ADL. Acupuncture vs. drugs (MD = −1.29, 95% CI: −1.77, −0.80; very low quality; Lee et al., 2009; MD=-2.80, 95% CI: −4.57, −1.02; low quality; Zhou et al., 2015; WMD = 1.99, 95% CI: 0.65, 3.34; moderate-quality) (Huang et al., 2019) improved the ADL. A combination of acupuncture and drugs vs. drugs alone (MD=-2.64, 95% CI: −4.95, −0.32; low quality) (Zhou et al., 2015) improved ADL. Exercise intervention (MD = 0.80, 95% CI: 0.42, 1.19; very low quality) (Rao et al., 2014) improved ADL.

#### ADAs-Cog

Only four MAs (Zhou et al., 2015, 2017; Dong et al., 2018; Huang et al., 2019) reported the effects of two interventions (acupuncture and rTMS) on ADAS-cog. Two reported superior effects of acupuncture than drugs (MD = −5.14, 95% CI: −8.75, −1.53; moderate-quality; Zhou et al., 2015; WMD = 5.56, 95% CI: 1.10, 6.03; low quality; Huang et al., 2019) with regards to ADAS-cog. tTMS compared with sham rTMS (MD = −3.56, 95% CI: −5.82, −1.48; moderate-quality) (Dong et al., 2018) decreased the ADAS-cog.

## Discussion

### Main Findings

In this overview, the different non-pharmacological interventions showed potential in reducing or improving the relevant outcomes, reflecting the clinical efficacy of these interventions. Exercise intervention showed the preliminary potential to improve ADL and MMSE. The cognitive intervention improved MMSE. rTMS may decrease ADAS-cog, and there was some evidence of improved MMSE, ADAS-cog, and ADL with acupuncture therapy. Efficacy was lacking for music therapy on the included outcomes for AD patients.

We used AMSTAR 2 and the GRADE system to assess the methodological quality and evidence quality of 10 MAs of non-pharmacological therapy for AD. The results for methodological quality by AMSTAR 2 showed that only two studies (Zhou et al., 2015; Dong et al., 2018) provided a protocol and had been registered. The lack of registration may result in a great adjustment of the research process as expected. Five of the MAs (Rao et al., 2014; Dong et al., 2018; Du et al., 2018; Huang et al., 2019; Jia et al., 2019) were screened and selected by two researchers, which was beneficial to accurate literature selection. The sources of funding are not reported in any of the included MAs, which may reduce the credibility of the research results due to potential conflicts of interest. Therefore, MAs of non-pharmacological therapy for AD should be registered and have a reported protocol before the study is carried out, and we should pay attention to any observed heterogeneity of the results of the review. The evidence quality of all the included outcomes was degraded. This overview of 10 studies and 24 outcomes showed that the quality of evidence was low and very low (58.3%); moderate evidence accounted for only 41.7%, and there was no high-quality evidence. Despite the potential efficacy of non-pharmacological interventions, the strength of evidence for all outcomes is still unsatisfactory.

### Role of Non-pharmacological Interventions

#### Exercise Intervention

According to a prospective cohort study, exercise is one of the healthy lifestyle factors that contribute to a lower risk of AD (Dhana et al., 2019). There is now strong evidence linking regular physical activity or exercise to higher cognitive function, decreased cognitive decline, and reduced risk of AD (Brown et al., 2013). Exercise may also play a role in maintaining/improving brain health by increasing the peripheral concentrations of blood brain-derived neurotrophic factor (BDNF) (Marinus et al., 2019). Research suggests that the mechanisms of physical exercise include enhancing Aβ clearance rates or reducing deposition and promoting hippocampal synaptic plasticity. It has been found that physical activity counteracts AD-associated declines in mitochondrial and immune system function (Intlekofer and Cotman, 2013). Physical exercise affects many mechanisms at the cellular and molecular levels. It improves the production of neurotrophic factors, neurotransmitters, and hormones and promotes neuronal survival and neuroplasticity (Mahalakshmi et al., 2020). However, evidence that short-term, single-component exercise interventions promote cognitive function and prevent cognitive decline or dementia in older adults is largely insufficient (Brasure et al., 2018). Our overview found that exercise intervention effectively improved MMSE (very low quality of evidence) and ADL (moderate quality of evidence). Exercise is an available intervention for AD that is worthy of further research and promotion.

#### Cognitive Intervention

Cognitive intervention is typically classified as cognitive stimulation, cognitive training, and cognitive rehabilitation. Enhanced brain plasticity may be a component of the mechanism underpinning the cognitive improvements associated with cognitive interventions (Jeong et al., 2016). However, the benefits on ADAS-Cog are generally not clinically significant, and difficulties with blinding of patients and the use of adequate placebo controls make comparison with the results of dementia drug treatments problematic (Huntley et al., 2015). Our overview found that cognitive intervention was effective for the MMSE, similar to the previous study above (Huntley et al., 2015).

#### Music Therapy

The results of study suggested that music therapy is effective in enhancing cognitive function and mental well-being, which can be recommended as an alternative approach to managing AD-associated symptoms (Lyu et al., 2018). Three independent, yet interconnected, mechanisms can be hypothesized to underlie the beneficial effect that music plays in enhancing memory in persons with AD. These include activation of dopaminergic pathways, sympathetic arousal, and default neuronal connectivity (Peck et al., 2016). However, although the evidence for short-term improvement in mood and reduction in behavioral disturbance is consistent, there are no high-quality longitudinal studies that demonstrated the long-term benefits of music therapy (McDermott et al., 2013). Our results concur with those from the cited studies.

#### rTMS

TMS is a non-invasive and painless technique that generates an electric current-inducing modulation in cortical excitability, which can stimulate and regulate the cortical function of the brain (Rabey and Dobronevsky, 2016). There is strong evidence that rTMS exerts significant neuroprotective and pro-cognitive effects in AD through the expression of hippocampal BDNF (Yulug et al., 2018). The study shows that rTMS can significantly improve cognitive ability in patients with mild to moderate AD. Stimulation of multiple sites and long-term treatment improved AD-associated cognitive performance, but heterogeneity among the studies was inevitable and may have influenced our results (Lin et al., 2019). Our overview found that rTMS was effective on ADAS-cog but not on MMSE.

#### Acupuncture Therapy

Acupuncture therapy is a unique non-pharmacologic therapy in Chinese medicine that may protect neurons from degeneration and promote axonal regeneration in neurodegenerative diseases such as AD (Li et al., 2014). Related research has demonstrated that acupuncture improves spatial learning and memory ability by ameliorating the dendritic structure (Kan et al., 2018). It is also suggested that acupuncture improves the cognitive function of AD by regulating glucose metabolism, enhancing neurotransmission, and reducing oxidative stress, Aβ protein deposition, and neuronal apoptosis. However, it is still challenging to clarify which specific signaling pathway contributes to the acupuncture effect (Cao et al., 2016). Recently, there has been increasing evidence that acupuncture may be an effective and safe way to treat AD. However, the results among SRs and MAs show varied and heterogeneous effects, making it difficult for clinicians and policymakers to conclude overall treatment efficacy (Lee et al., 2009; Zhou et al., 2015, 2017; Huang et al., 2019). Our overview found that acupuncture was effective for the MMSE, ADL, and ADAS-cog. We suggest that the role of acupuncture should be considered in the development of AD clinical practice guidelines.

### Suggestions for Future Research

The objective of any AD treatment is to achieve clinically meaningful symptom reduction in patients. Despite the promise of emerging therapies, existing pharmacological treatments dominate the AD treatment landscape (Ivashchenko et al., 2018), despite the lack of evidence on their effectiveness and inevitable side effects. For example, some antipsychotics are associated with an increased risk of stroke and death in individuals with dementia (Maust et al., 2015; Ralph and Espinet, 2018). In a subgroup of persons with AD, antipsychotics were associated with increased fracture (Watt et al., 2020). Given this, non-pharmacological therapy presents a potentially significant supplementary treatment for AD. The effectiveness of non-pharmacological therapy, including psychosocial interventions, for improving cognition or slowing down the progression of cognitive impairment in AD patients has been confirmed (Duan et al., 2018). When dealing with AD patients, it is suggested that clinicians should not forget non-pharmacological interventions.

“Determining the effectiveness of non-pharmacologic therapies can be difficult because of the large number of unique therapies tested, the diversity of therapeutic aims (from the improved overall quality of life to improvements in specific symptoms), the diverse stages of dementia represented (from mild to moderate to severe), the diverse types of dementia that may be present among participants in a particular study given the pervasiveness of mixed dementia, and the lack of standard methods for carrying out any individual therapy. With these multiple factors to consider, it is challenging to group together and compare non-pharmacologic therapies” (Alzheimer's Association, 2020). Therefore, future research needs to focus on the type and the form of non-pharmacological interventions that are most effective for the different severities of AD. Preliminary data suggest benefits on three outcomes related to cognitive function and quality of life. Furthermore, methodologically rigorous and adequately powered primary studies are necessary for each non-pharmacological intervention, reporting on consistently defined core outcomes in patients with AD.

### Strengths and Weaknesses of the Review

A comprehensive evaluation of the efficacy of multiple non-pharmacologic interventions in patients with AD is currently lacking, so we summarized and compared the determining efficacy of five non-pharmacologic therapies evaluated in 10 MAs. To our knowledge, this overview of MAs is the first to compare multiple non-pharmacologic strategies to facilitate decision making systematically. To reduce the risk of bias, we only included MAs of RCTs and excluded narrative reviews and reviews with non-RCTs and observational cohort studies. We assessed the quality of the reviews against the 16 domains of AMSTAR 2, which, compared with AMSTAR, has a wider range of applications and more scientific evaluation methods (Shea et al., 2007, 2017). We assessed the significant outcomes by the GRADE system to determine the strength of evidence. Most of the evidence of the included primary trials were acknowledged as of poor quality. We also identified the deficiencies of current studies and make recommendations for further research on non-pharmacological interventions for AD.

However, there are still some shortcomings. The number of included studies was limited, and some studies of non-pharmacological therapy for AD did not meet the inclusion criteria, such as plasmapheresis (Luengo-Matos et al., 2017), dance (Ruiz-Muelle and López-Rodríguez, 2019), light treatment (Mitolo et al., 2018), spaced retrieval training (Oren et al., 2014), and physical therapy interventions (Zhu et al., 2015). These studies were not evaluated, but their clinical effectiveness cannot be ruled out. We need more scientific and standard studies in the future. Besides that, only three main clinical outcomes (MMSE, ADL, and ADAS-cog) were included. The MMSE is a cognitive test that is the most common and widely used cognitive screening measure as part of the evaluation for AD (Folstein et al., 1975; Tsoi et al., 2015), and it has good validity and reliability (Razani et al., 2009). In AD, the decline in ADL is increasingly recognized as a source of considerable social, health, and economic costs (Alzheimer's Association, 2020), and ADAS-Cog (Rosen et al., 1984) is the most widely used general cognitive measure in clinical trials of AD. Research demonstrates that a three-point decline in ADAS-Cog may be an appropriate minimal clinical relevant change for clinical trials of early AD (Schrag and Schott, 2012). Unfortunately, despite the advantages, other outcomes reflecting AD status were not included, which need to be discussed further.

## Conclusion

Although there are gaps in the literature and a lack of high-quality evidence, the findings from this overview suggest that non-pharmacological interventions are important for AD patients. In the future, the quality of the original research needs to be improved, and strictly designed MAs should be carried out following methodological requirements. Higher-quality trials and patient-based MAs are needed to determine the benefits of specific non-pharmacological interventions.

## Data Availability Statement

The raw data supporting the conclusions of this article will be made available by the authors, without undue reservation.

## Author Contributions

L-YW and JP designed the study. L-YW and Y-JZ selected the relevant studies. Y-WC and Y-JZ extracted the data. L-YW and Y-WC performed the quality assessment. L-YW wrote the manuscript. All the authors contributed to the writing of this manuscript.

## Conflict of Interest

The authors declare that the research was conducted in the absence of any commercial or financial relationships that could be construed as a potential conflict of interest.

## Abbreviations

AD: Alzheimer's disease
ADAS-cog: Alzheimer's Disease Assessment Scale-cognitive section
ADL: activities of daily living
AMSTAR: A Measurement Tool to Assess Systematic Reviews
BDNF: brain-derived neurotrophic factor
GRADE: Grading of Recommendations, Assessment, Development and Evaluation
MAs: meta-analyses
MMSE: Mini-Mental State Examination
PICO: population, intervention, comparison, outcome
RCT: randomized clinical trial
rTMS: repetitive transcranial magnetic stimulation
SR: systematic review.

## References

[B1] Alves J. Magalhaes R. Thomas R. E. Goncalves O. F. Petrosyan A. Sampaio A. (2013). Is there evidence for cognitive intervention in Alzheimer disease? A systematic review of efficacy, feasibility, and cost-effectiveness. Alzheimer Dis Assoc Disord. 27, 195–203. 10.1097/WAD.0b013e31827bda55 23314062

[B2] Alzheimer's Association (2020). 2020 Alzheimer's disease facts and figures. Alzheimers Dement 16, 391–460. 10.1002/alz.12068 32157811

[B3] Alzheimer's Disease International (2015). World Alzheimer Report 2015—the Global Impact of Dementia: An Analysis of Prevalence Incidence, Cost and Trends. Available online at: https://www.alz.co.uk/research/world-report-2015 (accessed August, 2015).

[B4] Arvanitakis Z. Shah R. C. Bennett D. A. (2019). Diagnosis and management of dementia: review. JAMA. 322, 1589–1599. 10.1001/jama.2019.4782 31638686 PMC7462122

[B5] Bougioukas K. I. Liakos A. Tsapas A. Ntzani E. Haidich A. B. (2018). Preferred reporting items for overviews of systematic reviews including harms checklist: a pilot tool to be used for balanced reporting of benefits and harms. J Clin Epidemiol. 93:9–24. 10.1016/j.jclinepi.2017.10.002 29037888

[B6] Brasure M. Desai P. Davila H. Nelson V. A. Calvert C. Jutkowitz E.. (2018). Physical activity interventions in preventing cognitive decline and Alzheimer-type dementia: a systematic review. Ann Intern Med. 168, 30–38. 10.7326/m17-1528 29255839

[B7] Brown B. M. Peiffer J. J. Martins R. N. (2013). Multiple effects of physical activity on molecular and cognitive signs of brain aging: can exercise slow neurodegeneration and delay Alzheimer's disease? Mol Psychiatry. 18, 864–874. 10.1038/mp.2012.162 23164816

[B8] Cammisuli D. M. Innocenti A. Fusi J. Franzoni F. Pruneti C. (2018). Aerobic exercise effects upon cognition in Alzheimer's Disease: a systematic review of randomized controlled trials. Arch Ital Biol. 156, 54–63. 10.12871/00039829201816 30039836

[B9] Cao Y. Zhang L. W. Wang J. Du S. Q. Xiao L. Y. Tu J. F.. (2016). Mechanisms of acupuncture effect on Alzheimer's disease in animal- based researches. Curr Top Med Chem. 16, 574–578. 10.2174/1568026615666150813144942 26268326

[B10] Chan K. Y. Wang W. Wu J. J. Liu L. Theodoratou E. Car J.. (2013). Epidemiology of Alzheimer's disease and other forms of dementia in China, 1990-2010: a systematic review and analysis. Lancet. 381, 2016–2023. 10.1016/s0140-6736(13)60221-4 23746902

[B11] Dhana K. Evans D. A. Rajan K. B. Bennett D. A. Morris M. C. (2019). Impact of healthy lifestyle factors on the risk of Alzheimer's dementia: findings from two prospective cohort studies. Alzheimer's and Dementia. 15:P207 10.1016/j.jalz.2019.06.4547

[B12] Dong X. Yan L. Huang L. Guan X. Dong C. Tao H.. (2018). Repetitive transcranial magnetic stimulation for the treatment of Alzheimer's disease: a systematic review and meta-analysis of randomized controlled trials. PLoS ONE. 13:e0205704. 10.1371/journal.pone.0205704 30312319 PMC6185837

[B13] Du Z. Li Y. Li J. Zhou C. Li F. Yang X. (2018). Physical activity can improve cognition in patients with Alzheimer's disease: a systematic review and meta-analysis of randomized controlled trials. Clin Interv Aging. 13, 1593–1603. 10.2147/cia.S169565 30233156 PMC6130261

[B14] Duan Y. Lu L. Chen J. Wu C. Liang J. Zheng Y.. (2018). Psychosocial interventions for Alzheimer's disease cognitive symptoms: a Bayesian network meta-analysis. BMC Geriatr. 18:175. 10.1186/s12877-018-0864-6 30086714 PMC6081912

[B15] Dubois B. Feldman H. H. Jacova C. DeKosky S. T. Gateau P. B. Cummings J.. (2007). Research criteria for the diagnosis of Alzheimer's disease: revising the NINCDS-ADRDA criteria. Lancet Neurol. 6, 734–746. 10.1016/S1474-4422(07)70178-3 17616482

[B16] Folstein M. F. Folstein S. E. McHugh P. R. (1975). Mini-mental state. A practical method for grading the cognitive state of patients for the clinician. J Psychiatr Res. 12, 189–198. 10.1016/0022-3956(75)90026-6 1202204

[B17] García-Casares N. Moreno-Leiva R. M. García-Arnés J. A. (2017). Music therapy as a non-pharmacological treatment in alzheimer's disease. A systematic review. Rev. Neurol. 65, 529–538. Available online at: http://www.embase.com/search/results?subaction=viewrecord&from=export&id=L623517089 29235615

[B18] Gordon M. Guyatt G. (2020). Assessment of evidence quality in IBD guidance: the use and misuse of GRADE. Gastroenterology 159, 1209–1215. 10.1053/j.gastro.2020.06.092 32681924

[B19] Guyatt G. H. Oxman A. D. Vist G. E. Kunz R. Falck-Ytter Y. Alonso-Coello P.. (2008). GRADE: an emerging consensus on rating quality of evidence and strength of recommendations. BMJ 336, 924–926. 10.1136/bmj.39489.470347.AD 18436948 PMC2335261

[B20] Haapasalo A. Pikkarainen M. Soininen H. (2015). Alzheimer's disease: a report from the 7th Kuopio Alzheimer symposium. Neurodegener Dis Manag. 5, 379–382. 10.2217/nmt.15.31 26477468

[B21] Huang Q. Luo D. Chen L. Liang F. X. Chen R. (2019). Effectiveness of acupuncture for Alzheimer's disease: an updated systematic review and meta-analysis. Curr Med Sci. 39, 500–511. 10.1007/s11596-019-2065-8 31209824

[B22] Huntley J. D. Gould R. L. Liu K. Smith M. Howard R. J. (2015). Do cognitive interventions improve general cognition in dementia? A meta-analysis and meta-regression. BMJ Open. 5, e005247. 10.1136/bmjopen-2014-005247 25838501 PMC4390716

[B23] Intlekofer K. A. Cotman C. W. (2013). Exercise counteracts declining hippocampal function in aging and Alzheimer's disease. Neurobiol Dis. 57:47–55. 10.1016/j.nbd.2012.06.011 22750524

[B24] Ivashchenko A. V. Brooks L. Tran Z. V. (2018). Differential effects of pharmacologic and non-pharmacologic threatments of Alzheimer's disease: a comprehensive summary of evidence and meta-amalysis. Alzheimer's Dementia 14, P676–P677. 10.1016/j.jalz.2018.06.710

[B25] Jeong J. H. Na H. R. Choi S. H. Kim J. Na D. L. Seo S. W.. (2016). Group- and home-based cognitive intervention for patients with mild cognitive impairment: a randomized controlled trial. Psychother Psychosom. 85, 198–207. 10.1159/000442261 27230861

[B26] Jia R. X. Liang J. H. Xu Y. Wang Y. Q. (2019). Effects of physical activity and exercise on the cognitive function of patients with Alzheimer disease: a meta-analysis. BMC Geriatr. 19:181. 10.1186/s12877-019-1175-2 31266451 PMC6604129

[B27] Kan B. H. Yu J. C. Zhao L. Zhao J. Li Z. Suo Y. R.. (2018). Acupuncture improves dendritic structure and spatial learning and memory ability of Alzheimer's disease mice. Neural Regen Res. 13, 1390–1395. 10.4103/1673-5374.235292 30106051 PMC6108219

[B28] Kishita N. Backhouse T. Mioshi E. (2020). Nonpharmacological interventions to improve depression, anxiety, and quality of life (QoL) in people with dementia: an overview of systematic reviews. J Geriatr Psychiatry Neurol. 33, 28–41. 10.1177/0891988719856690 31203712

[B29] Lee M. S. Shin B. C. Ernst E. (2009). Acupuncture for Alzheimer's disease: a systematic review. Int J Clin Pract. 63, 874–879. 10.1111/j.1742-1241.2009.02043.x 19490197

[B30] Li X. Guo F. Zhang Q. Huo T. Liu L. Wei H.. (2014). Electroacupuncture decreases cognitive impairment and promotes neurogenesis in the APP/PS1 transgenic mice. BMC Complement Altern Med. 14:37. 10.1186/1472-6882-14-37 24447795 PMC3907495

[B31] Lin Y. Jiang W. J. Shan P. Y. Lu M. Wang T. Li R. H.. (2019). The role of repetitive transcranial magnetic stimulation (rTMS) in the treatment of cognitive impairment in patients with Alzheimer's disease: a systematic review and meta-analysis. J Neurol Sci. 398:184–191. 10.1016/j.jns.2019.01.038 30735817

[B32] Lorenz R. C. Matthias K. Pieper D. Wegewitz U. Morche J. Nocon M.. (2019). A psychometric study found AMSTAR 2 to be a valid and moderately reliable appraisal tool. J Clin Epidemiol. 114, 133–140. 10.1016/j.jclinepi.2019.05.028 31152864

[B33] Luengo-Matos S. Polo-Desantos M. Pablo J. Orrego C. Sanchez-Gomez L. M. (2017). Assessment of plasmapheresis for Alzheimer's disease systematic review. Int J Technol Assess Health Care. 33:227 10.1017/S026646231700404428641608

[B34] Lyu J. Zhang J. Mu H. Li W. Champ M. Xiong Q.. (2018). The effects of music therapy on cognition, psychiatric symptoms, and activities of daily living in patients with Alzheimer's disease. J Alzheimers Dis. 64, 1347–1358. 10.3233/jad-180183 29991131

[B35] Mahalakshmi B. Maurya N. Lee S. D. Bharath Kumar V. (2020). Possible neuroprotective mechanisms of physical exercise in neurodegeneration. Int J Mol Sci. 21, E5895. 10.3390/ijms21165895 32824367 PMC7460620

[B36] Maresova P. Dolejs J. Kuca K. (2018). Call for a uniform strategy of collecting Alzheimer's disease costs: a review and meta-analysis. J Alzheimers Dis. 63, 227–238. 10.3233/jad-171028 29578487

[B37] Marinus N. Hansen D. Feys P. Meesen R. Timmermans A. Spildooren J. (2019). The impact of different types of exercise training on peripheral blood brain-derived neurotrophic factor concentrations in older adults: a meta-analysis. Sports Med. 49, 1529–1546. 10.1007/s40279-019-01148-z 31270754

[B38] Maust D. T. Kim H. M. Seyfried L. S. Chiang C. Kavanagh J. Schneider L. S.. (2015). Antipsychotics, other psychotropics, and the risk of death in patients with dementia: number needed to harm. JAMA Psychiatry 72, 438–445. 10.1001/jamapsychiatry.2014.3018 25786075 PMC4439579

[B39] McDermott O. Crellin N. Ridder H. M. Orrell M. (2013). Music therapy in dementia: a narrative synthesis systematic review. Int J Geriatr Psychiatry 28, 781–794. 10.1002/gps.3895 23080214

[B40] McKhann G. Drachman D. Folstein M. Katzman R. Price D. Stadlan E. M. (1984). Clinical diagnosis of Alzheimer's disease: report of the NINCDS-ADRDA Work Group under the auspices of Department of Health and Human Services Task Force on Alzheimer's Disease. Neurology 34, 939–944. 10.1212/wnl.34.7.939 6610841

[B41] Mitolo M. Tonon C. La Morgia C. Testa C. Carelli V. Lodi R. (2018). Effects of light treatment on sleep, cognition, mood, and behavior in Alzheimer's disease: a systematic review. Dement Geriatr Cogn Disord. 46, 371–384. 10.1159/000494921 30537760

[B42] O'Brien J. T. Holmes C. Jones M. Jones R. Livingston G. McKeith I.. (2017). Clinical practice with anti-dementia drugs: a revised (third) consensus statement from the British Association for Psychopharmacology. J Psychopharmacol. 31, 147–168. 10.1177/0269881116680924 28103749

[B43] Olazaran J. Reisberg B. Clare L. Cruz I. Pena-Casanova J. Del Ser T.. (2010). Nonpharmacological therapies in Alzheimer's disease: a systematic review of efficacy. Dement Geriatr Cogn Disord. 30, 161–178. 10.1159/000316119 20838046

[B44] Oren S. Willerton C. Small J. (2014). Effects of spaced retrieval training on semantic memory in Alzheimer's disease: a systematic review. J Speech Lang Hear Res. 57, 247–270. 10.1044/1092-4388(2013/12-0352) 24023380

[B45] Peck K. J. Girard T. A. Russo F. A. Fiocco A. J. (2016). Music and memory in Alzheimer's disease and the potential underlying mechanisms. J Alzheimers Dis. 51, 949–959. 10.3233/JAD-150998 26967216

[B46] Pedroza P. Chakrabarti S. Chapin A. Liu A. Matyasz T. Dieleman J. L. (2019). Costs of Alzheimer's disease and dementia in 188 countries. Alzheimer's Dementia 15:P1635 10.1016/j.jalz.2019.06.4877

[B47] Rabey J. M. Dobronevsky E. (2016). Repetitive transcranial magnetic stimulation (rTMS) combined with cognitive training is a safe and effective modality for the treatment of Alzheimer's disease: clinical experience. J Neural Transm. 123, 1449–1455. 10.1007/s00702-016-1606-6 27631152

[B48] Ralph S. J. Espinet A. J. (2018). Increased all-cause mortality by antipsychotic drugs: updated review and meta-analysis in dementia and general mental health care. J Alzheimers Dis Rep. 2, 1–26. 10.3233/adr-170042 30480245 PMC6159703

[B49] Rao A. K. Chou A. Bursley B. Smulofsky J. Jezequel J. (2014). Systematic review of the effects of exercise on activities of daily living in people with Alzheimer's disease. Am J Occup Ther. 68, 50–56. 10.5014/ajot.2014.009035 24367955 PMC5360200

[B50] Razani J. Wong J. T. Dafaeeboini N. Edwards-Lee T. Lu P. Alessi C.. (2009). Predicting everyday functional abilities of dementia patients with the Mini-Mental State Examination. J Geriatr Psychiatry Neurol. 22, 62–70. 10.1177/0891988708328217 19196632 PMC2679691

[B51] Rosen W. G. Mohs R. C. Davis K. L. (1984). A new rating scale for Alzheimer's disease. Am J Psychiatry. 141, 1356–1364. 10.1176/ajp.141.11.1356 6496779

[B52] Ruiz-Muelle A. López-Rodríguez M. M. (2019). Dance for people with Alzheimer's disease: a systematic review. Curr Alzheimer Res. 16, 919–933. 10.2174/1567205016666190725151614 31345149

[B53] Sá C. C. da Silva D. F. Bigongiari A. Machado-Lima A. (2019). Efficacy of cognitive rehabilitation in improving and maintaining daily living activities in patients with alzheimer's disease: a systematic review of literature. Jornal Brasileiro de Psiquiatria. 68, 153–160. 10.1590/0047-2085000000241

[B54] Schrag A. Schott J. M. (2012). What is the clinically relevant change on the ADAS-Cog? J Neurol Neurosurg Psychiatry. 83, 171–173. 10.1136/jnnp-2011-300881 22019547

[B55] Shea B. J. Grimshaw J. M. Wells G. A. Boers M. Andersson N. Hamel C.. (2007). Development of AMSTAR: a measurement tool to assess the methodological quality of systematic reviews. BMC Med Res Methodol. 7:10. 10.1186/1471-2288-7-10 17302989 PMC1810543

[B56] Shea B. J. Reeves B. C. Wells G. Thuku M. Hamel C. Moran J. (2017). AMSTAR 2: a critical appraisal tool for systematic reviews that include randomised or non-randomised studies of healthcare interventions, or both. BMJ 358:j4008 10.1136/bmj.j4008 28935701 PMC5833365

[B57] Tsoi K. K. Chan J. Y. Hirai H. W. Wong S. Y. Kwok T. C. (2015). Cognitive tests to detect dementia: a systematic review and meta-analysis. JAMA Intern Med. 175, 1450–1458. 10.1001/jamainternmed.2015.2152 26052687

[B58] Wang Y. Zhen T. Liao Y. Li L. Zhang Y. (2020). A meta-analysis of the effect of music therapy on alzheimer's disease. Int J Clin Exp Med. 13, 317–329. Available online at: http://www.embase.com/search/results?subaction=viewrecord&from=export&id=L2003914068

[B59] Watt J. A. Goodarzi Z. Veroniki A. A. Nincic V. Khan P. A. Ghassemi M.. (2020). Safety of pharmacologic interventions for neuropsychiatric symptoms in dementia: a systematic review and network meta-analysis. BMC Geriatr. 20:212. 10.1186/s12877-020-01607-7 32546202 PMC7298771

[B60] Xu J. Murphy S. L. Kockanek K. D. Arias E. (2020). Mortality in the United States, 2018. NCHS Data Brief. 355:1–8 32487294

[B61] Yulug B. Hanoglu L. Khanmammadov E. Duz O. A. Polat B. Hanoglu T.. (2018). Beyond the therapeutic effect of rTMS in Alzheimer's disease: a possible neuroprotective role of hippocampal BDNF?: A minireview. Mini Rev Med Chem. 18, 1479–1485. 10.2174/1389557517666170927162537 28971775

[B62] Zhou J. Peng W. N. Xu M. Li W. Liu Z. S. (2015). The effectiveness and safety of acupuncture for patients with Alzheimer disease a systematic review and meta-analysis of randomized controlled trials. Medicine 94:e933. 10.1097/md.0000000000000933 26039131 PMC4616366

[B63] Zhou S. Dong L. He Y. Xiao H. (2017). Acupuncture plus herbal medicine for Alzheimer's disease: a systematic review and meta-analysis. Am J Chin Med. 45, 1327–1344. 10.1142/s0192415x17500732 28922926

[B64] Zhu X. C. Yu Y. Wang H. F. Jiang T. Cao L. Wang C.. (2015). Physiotherapy intervention in Alzheimer's disease: systematic review and meta-analysis. J Alzheimers Dis. 44, 163–174. 10.3233/jad-141377 25201787

[B65] Zucchella C. Sinforiani E. Tamburin S. Federico A. Mantovani E. Bernini S.. (2018). The multidisciplinary approach to Alzheimer's disease and dementia. A narrative review of non-pharmacological treatment. Front Neurol. 9:1058. 10.3389/fneur.2018.010 30619031 PMC6300511

