# Supplementary material for: Overview of Meta-Analyses of Five Non-pharmacological Interventions for Alzheimer's Disease
Source: Front Aging Neurosci. 2020 Nov 25;12:594432. doi: 10.3389/fnagi.2020.594432 (PMC7723835; doi:10.3389/fnagi.2020.594432)
Supplement: Supplementary file 1 [file Table_1.DOCX]

Supplementary Material

# Supplementary Table

**Search Strategy**

(1) PubMed

| **Search** | **Query** | **Items** |
| --- | --- | --- |
| #1 | "Alzheimer Disease"[Mesh] | 92603 |
| #2 | (Alzheimer Disease[Title/Abstract]) OR Senile Dementia[Title/Abstract] | 19986 |
| #3 | #1OR#2 | 100082 |
| #4 | "Meta-Analysis" [Publication Type] OR "Meta-Analysis as Topic"[Mesh] | 132041 |
| #5 | (Meta analys*[Title/Abstract]) OR Systematic review*[Title/Abstract] | 263946 |
| #6 | # 4OR #5 | 289206 |
| #7 | #3 AND #6 | 1908 |

(2) Embase

| **Search** | **Query** | **Items** |
| --- | --- | --- |
| #1 | 'alzheimer disease'/exp | 197786 |
| #2 | 'alzheimer disease':ab,ti OR 'senile dementia':ab,ti | 25244 |
| #3 | #1 OR #2 | 202616 |
| #4 | 'meta analysis'/exp | 184596 |
| #5 | 'meta analys*':ab,ti OR 'systematic review*':ab,ti | 332322 |
| #6 | #4 OR #5 | 371106 |
| #7 | #3 OR #6 | 3853 |

(3) Cochrane Library

| **Search** | **Query** | **Items** |
| --- | --- | --- |
| #1 | MeSH descriptor: [Alzheimer Disease] explode all trees | 3307 |
| #2 | ("Alzheimer Disease"):ti,ab,kw OR ("Senile Dementia"):ti,ab,kw | 9896 |
| #3 | #1 OR #2 | 9896 |
| #4 | MeSH descriptor: [Meta-Analysis as Topic] explode all trees | 297 |
| #5 | ("Meta analys*"):ti,ab,kw OR ("Systematic review*"):ti,ab,kw | 10900 |
| #6 | #4 OR #5 | 11168 |
| #7 | #3 AND #6 | 115 |

(4) Web of Science

| **Search** | **Query** | **Items** |
| --- | --- | --- |
| #1 | TS: ("Alzheimer Disease") OR TS: ("Senile Dementia"):ti,ab,kw | 29269 |
| #2 | TS: ("Meta analys*") OR TS: ("Systematic review*"):ti,ab,kw | 300842 |
| #3 | #1 AND #2 | 628 |
