# Supplementary material for: Overview of Meta-Analyses of Five Non-pharmacological Interventions for Alzheimer's Disease
Source: Front Aging Neurosci. 2020 Nov 25;12:594432. doi: 10.3389/fnagi.2020.594432 (PMC7723835; doi:10.3389/fnagi.2020.594432)
Supplement: Supplementary file 3 [file Table_3.docx]

Supplementary Material

# Supplementary Table

**The key parameters of five interventions**

**(1) Acupuncture therapy**

| **Study** | **Electronic** | **Time (min)** | **Frequency** | **Course (week)** | **Acupoints** |
| --- | --- | --- | --- | --- | --- |
| Lee 2009 | Electronic acupuncture (continuous wave; 2-4HZ) | 15-30 | once day, 6 days/week | 8 | GV20、EX-HN1、GB20、BL23、GV14、HT7、PC6、SP6、KI3、ST36、ST40、LR3、GV29 |
| Zhou 2017 | / | 30 | once day, 5-6 days/week | 4-24 | ST36、EX-HN1、GV20、GV24、PC6、SP6、HT7、KI3、GB20、GV16、TE5、ST9、GV23、GV15、GB12、BL10、GV29、GV14、CV4、LI4、LR3、KI6、KI4、GB39、ST40、CV6、BL14、BL15、BL17、BL18、BL23 |
| Zhou 2015 | 3 of these 10 RCTs used electroacupuncture | / | / | 3-24 | GV20、ST 36、SP10、EX-HN1、KI3、GV14、CV17、CV12、CV6、TE5、GB39、PC6、BL23、GV29、SP6、KI4 |
| Huang 2019 | 2 of these 13 RCTs used electroacupuncture | 30-60 | once day, 5-6 days/week | 4-16 | / |

Note: These included MAs used manual acupuncture and electroacupuncture (continuous wave, 2-4HZ). The three most frequently used acupoints are EX-HN1, GV20 and ST36. Needle retention time is 30 minutes. The course of acupuncture therapy is 8-12 weeks with the stimulation frequency of once day, 5-6 days/week.

**(2) Exercise intervention**

| **Study** | **Type** | **Minutes per session** | **Frequency** | **Duration** |
| --- | --- | --- | --- | --- |
| Jia 2019 | Function-task physical activity; daily exercise and walking; treadmill walking; brisk walking; aerobic exercise; cycle ergometer exercise | 30-60 | 1-3h/week, 1-7 days/ week | 12-24 weeks |
| Rao 2014 | walking programs; functionalactivity performance; stretching, strength and resistance training; balance exercises; and aerobic and endurance training | / | / | 12-48 weeks |
| Du 2018 | Daily exercise and walking; A daily exercise program: aerobic fitness, strength training, and balance and flexibility training; cycling train; aerobic exercise | 30-60min/day | 2-4 times/week | 6-24 weeks |

Note: The type of exercise intervention mainly includes treadmill/brisk walking, cycle ergometer exercise, aerobic exercise, strength training, and balance and flexibility training. Intervention time ranges from 6-24 weeks.

**(3) Cognitive intervention**

| **Study** | **Type** | **Time** | **Frequency** | **Duration** |
| --- | --- | --- | --- | --- |
| Alves 2013 | Memory-Training Program; Attention-Stimulation Activities; Cognitive Training; Cognitive stimulation therapy | 45min-1h | 1-2/week | 5-10 weeks |

Note: The followings are the different type of intervention of included MA: memory-training program, attention-stimulation activities, cognitive training, and cognitive stimulation therapy. The duration of cognitive intervention is 5-10 weeks with the frequency of 1~2 times a week, 45~60min each time.

**(4) Music therapy**

| **Study** | **Intervention personnel** | **Intervention mode** | **Intervention time** |
| --- | --- | --- | --- |
| Wang 2020 | Music therapist; Trained doctors and nurses; professional singers and pianists; Rehabilitation instructor | Passive music therapy; Active music therapy; Combination of active and passive music therapy | 1-12 months |

Note: There are three modes of music therapy, active music therapy that includes singing, dancing, or instrument performance, passive music therapy which involves listening to live or recorded music, and combination of active and passive music therapy. It is expected to be more effective than passively listening to music as active music therapy involves cognitive training or even physical exercise (as the popular senior people’s square dance “Guanchang Wu” in China).

**(5) Repetitive transcranial magnetic stimulation (rTMS)**

| **Study** | **Stimulation target** | **Stimulation protocol** | | | |
| --- | --- | --- | --- | --- | --- |
| Dong 2018 | L/R DLPFC; Bilateral DLPFC; Broca; Wernicke; L/R pSAC | 10-20HZ | 80-100% RMT | 1200-2000 pulses per session | 5-30sessions over 2-6 weeks |

R, right; L, left; DLPFC, dorsolateral prefrontal cortex; pSAC, parietal somatosensory association cortex; RMT, resting motor threshold

Note: Dorsolateral prefrontal cortex is the most common used stimulation target of rTMS. And the stimulation protocol is as following: 20HZ, 90-110% resting motor threshold, 2000 pluses per session, 5 sessions per week for 2-6 weeks.
